# Novel Functional Grape Juices Fortified with Free or Immobilized *Lacticaseibacillus rhamnosus* OLXAL-1

**DOI:** 10.3390/microorganisms11030646

**Published:** 2023-03-02

**Authors:** Anastasios Nikolaou, Gregoria Mitropoulou, Grigorios Nelios, Yiannis Kourkoutas

**Affiliations:** Laboratory of Applied Microbiology and Biotechnology, Department of Molecular Biology & Genetics, Democritus University of Thrace, 68100 Alexandroupolis, Greece

**Keywords:** *Lacticaseibacillus rhamnosus* OLXAL-1, Type-1 diabetes, probiotics fortification, grape juice, biopreservatives, minor volatiles

## Abstract

During the last decade, a rising interest in novel functional products containing probiotic microorganisms has been witnessed. As food processing and storage usually lead to a reduction of cell viability, freeze-dried cultures and immobilization are usually recommended in order to maintain adequate loads and deliver health benefits. In this study, freeze-dried (free and immobilized on apple pieces) *Lacticaseibacillus rhamnosus* OLXAL-1 cells were used to fortify grape juice. Juice storage at ambient temperature resulted in significantly higher (>7 log cfu/g) levels of immobilized *L. rhamnosus* cells compared to free cells after 4 days. On the other hand, refrigerated storage resulted in cell loads > 7 log cfu/g for both free and immobilized cells for up to 10 days, achieving populations > 10^9^ cfu per share, with no spoilage noticed. The possible resistance of the novel fortified juice products to microbial spoilage (after deliberate spiking with *Saccharomyces cerevisiae* or *Aspergillus niger*) was also investigated. Significant growth limitation of both food-spoilage microorganisms was observed (both at 20 and 4 °C) when immobilized cells were contained compared to the unfortified juice. Keynote volatile compounds derived from the juice and the immobilization carrier were detected in all products by HS-SPME GC/MS analysis. PCA revealed that both the nature of the freeze-dried cells (free or immobilized), as well as storage temperature affected significantly the content of minor volatiles detected and resulted in significant differences in the total volatile concentration. Juices with freeze-dried immobilized cells were distinguished by the tasters and perceived as highly novel. Notably, all fortified juice products were accepted during the preliminary sensory evaluation.

## 1. Introduction

Development of functional foods has been a matter of intense scientific and commercial interest for several decades now [1,2]. The term “functional foods” includes a wide variety of products consisting of various components (e.g., nutraceuticals, prebiotics, vitamins, bioactive compounds, probiotics, etc.) [3] that may potentially confer positive effects on the consumers’ body functions, reduce the risk of a disease, or promote well-being in general [4]. A vast part of the functional foods’ market consists of products fermented or enriched with probiotic microorganisms, mainly but not exclusively, of the *Lactobacillus* and *Bifidobacteria* species [5,6]. Nevertheless, according to FAO/WHO, “probiotics are microorganisms (bacteria or yeasts) which, when administered in adequate concentrations, provide health benefits to the host” [7].

Recently, a novel wild-type *Lacticaseibacillus rhamnosus* OLXAL-1, isolated from olives [8], demonstrated significant antidiabetic capability to alleviate Type-1 diabetes symptoms, an illness that has dramatically increased in developed countries over the past decades.

While the majority of probiotic products traditionally relies on dairy [9], due to modern lifestyle and health reasons (e.g., lactose intolerance, milk allergies, high cholesterol, veganism, etc.), there is an increasing consumer interest in alternatives like fruit juices [10,11,12,13]. Fruit juices are very popular, eagerly consumed and contain significant amounts of dietary fibers, antioxidants, polyphenols, minerals, enzymes and vitamins, while the addition of probiotics may further enhance their benefits and value [14,15]. Thus, an upsurge in the development of non-dairy functional beverages, like fruit juices, has been noticed [9,16,17,18,19,20,21,22,23,24]. In fact, grapes and grape juice (in particular), are part of a healthy diet in many countries, and could potentially be exploited for functional food development [25,26,27]. Other than being highly nutrient, the fruit juices’ matrix may also provide a suitable environment for probiotics growth and survival [16]. This is a very important matter, as probiotic microorganisms must survive the entire food processing chain (manufacture, storage, serving) and retain adequate numbers (at least 10^7^ cfu/mL at the time of consumption), in order to deliver their functional features [1]. 

For that reason, cell immobilization technology is suggested in the production of novel functional foods, as it is known to enhance probiotic survival and thus result in longer preservation times, protection against microbial contamination, etc. [28]. The selection of a suitable immobilization carrier (e.g., fruit pieces) is however a matter of high importance, too, as it affects the cell adhesion and the colonization properties of functional cells [29,30] and could be utilized for the production of symbiotic (prebiotic + probiotic) functional components [31]. Likewise, freeze-drying technology is recommended, as it results in the maintenance of cell viability and operational stability, extends product shelf life, creates easy-to-handle and transport conditions, diminishes storage costs, etc. [32].

In general, fruit juices, due to their low pH values, do not favor the growth of spoilage and pathogenic microorganisms, making them rather safe and attractive to consumers [33]. Nevertheless, yeasts and molds can be considered the major reasons for fruit juice spoilage. They can grow in harsh environments with low pH, low water activity, and high sugar content. *Saccharomyces cerevisiae* and *Aspergillus niger* represent the most common spoilage microorganisms in fruit juices [34]. To overcome this problem, apart from pasteurization, the use of chemical additives (such as nitrite, sodium chloride, and organic acids) is a common practice in the food industry [35]. Due to consumers’ awareness, though, today there is mounting pressure on food manufacturers to either completely avoid the use of chemical preservatives or to adopt “natural” alternatives [36]. Functional cultures and microbial derivatives seem to play a significant role in the prevention of food-spoilage. Biopreservation uses the antimicrobial potential of some microorganisms to prevent spoilage and pathogenic microbe growth in foods [37]. The majority of biopreservation research has been focused on lactic acid bacteria’s antagonistic activities against spoilage and pathogenic microorganisms [38]. The antagonistic activities of lactic acid bacteria against other microbes in foods have been related to several mechanisms, such as the production of organic acids, H_2_O_2_, antibacterial bacteriocins, antimicrobial metabolites, such as diacetyl and reuterin and the reduction of pH [39]. 

In the present study, a novel juice product fortified with *Lacticaseibacillus rhamnosus* OLXAL-1 cells (previously evaluated for their antidiabetic properties) was developed. Data indicating the effective survival of *L. rhamnosus* through storage (at 20 °C and 4 °C) and possible resistance against food-spoilage microorganisms (*Saccharomyces cerevisiae* or *Aspergillus niger*), are presented. 

## 2. Materials and Methods

### 2.1. Microbial Cultures

*Lacticaseibacillus rhamnosus* OLXAL-1 [8], *Saccharomyces cerevisiae* Uvaferm NEM (Lallemand, Montreal, QC, Canada), and *Aspergillus niger* 19111 were used in this study.

*L. rhamnosus* OLXAL-1 was grown on a synthetic medium (2.0% *w*/*v* glucose, 0.2% *w/v* KH_2_PO_4_, 0.03% *w/v* MgSO_4_, 0.6% CH_3_COONa, 2.5% *w/v* yeast extract, 0.1% *v*/*v* Tween 80 and 0.005% *w*/*v* MnSO_4_) at 37 °C for 24 h.

*Saccharomyces cerevisiae* Uvaferm NEM was grown on Yeast extract Peptone Dextrose (YPD) broth (yeast extract 10 g/L, peptone 20 g/L, dextrose 20 g/L) at 28 °C for 24 h. 

*Aspergillus niger* 19111 was grown on Malt Agar (Condalab) at 37 °C for 7 days. Prior to use, all culture media were sterilized at 121 °C for 20 min.

### 2.2. Cell Immobilization and Production of Freeze-Dried Cultures 

Grown *L. rhamnosus* OLXAL-1 cells were harvested by centrifugation (8000× *g* for 15 min at 4 °C), rinsed with sterile ¼ Ringer’s solution (VWR International GmbH, Radnor, PA, USA) and subsequently centrifuged again (wet free cells). 

For the immobilization process, rinsed and harvested cells were resuspended in sterile ¼ Ringer’s solution up to the initial culture volume (immobilization solution). Apple pieces (0.4 ± 0.1 cm side length) were then submerged in the immobilization solution (in a ratio of 60% *w*/*v*) and left undisturbed for 4 h at 20 °C. After the immobilization process was completed, apple pieces were strained and rinsed with sterile ¼ Ringer’s solution, in order to remove any free non-immobilized cells (wet immobilized cells).

Freeze-dried immobilized cells were prepared on a BenchTop Pro (Virtis, SP Scientific, Warminster, PA, USA), as recently described [40]. For comparison reasons, free *L. rhamnosus* OLXAL-1 cells were also subjected to freeze-drying. 

Wet and freeze-dried immobilized or free cells were finally stored at room (20 °C) or refrigeration (4 °C) temperatures and their counts were monitored at various intervals.

### 2.3. Novel Juice Products

Concentrated grape juice of the Muscat Hamburg variety (Tyrnavos Cooperative Winery and Distillery, Tyrnavos, Greece) was diluted with sterilized deionized water to a final ~140 g/L. Freeze-dried immobilized cells on apple pieces were directly incorporated in grape juice (reaching a proportion of 20% *w*/*v* in the reconstituted juice product). For comparison reasons, juice products fortified with free freeze-dried *L. rhamnosus* OLXAL-1 cells (~0.033% *w*/*v*) were also prepared. Juice products containing only freeze-dried apple pieces or no cultures at all were used as controls. 

All products were stored at room (20 °C) or refrigeration (4 °C) temperatures for 14 and 30 days, respectively, in order to determine the product’s shelf life. 

### 2.4. Susceptibility to Spoilage 

Novel juice products were deliberately inoculated either with *S. cerevisiae* (inoculum of 10^4^ cfu/mL) or *Aspergillus niger* (inoculum of 10^4^ spores/mL) and their levels were monitored during storage at room (20 °C) or refrigeration temperatures (4 °C). Juice products without *L. rhamnosus* OLXAL-1 cells (free or immobilized) were used as control samples. 

### 2.5. Microbiological Analyses 

#### 2.5.1. *L. rhamnosus* OLXAL-1 Cell Counts 

Levels of free and immobilized cells were determined as recently described [41]. In brief, 5 g of immobilized cultures were blended with 45 mL sterile ¼ Ringer’s solution. Accordingly, 1 mL of free cell culture was transferred to 9 mL of sterile ¼ Ringer’s solution. Decimal serial dilutions in ¼ Ringer’s solution were performed, followed by plate counting on MRS agar plates after incubation at 37 °C for 72 h. Cell loads were expressed as log cfu/g immobilization carrier or log cfu/mL culture.

The survival rates of freeze-dried *L. rhamnosus* OLXAL-1 cells during storage were calculated as recently demonstrated [8].

In order to determine *L. rhamnosus* OLXAL-1 counts, 50 g of the novel juice products were homogenized with an iMix bag mixer (Interlab, Mourjou, France), serially diluted and subsequently plated on MRS Agar (Condalab, Madrid, Spain).

#### 2.5.2. Populations of Food-Spoilage Microorganisms 

In juice products deliberately spiked with food-spoilage yeast/fungi, populations were determined as follows:

- *S. cerevisiae* counts were determined on YPD Agar (yeast extract 10 g/L, peptone 20 g/L, dextrose 20 g/L, agar 20 g/L) after incubation at 28 °C for 72 h.
- *A. niger* spores were determined after enumeration on Neubauer plate (spores/g). *A. niger* counts (log cfu/mL) were determined on Malt Agar after incubation for 72 h at 37 °C [34].

#### 2.5.3. Microbial Contaminants

The presence of other foodborne microorganisms during storage of freeze-dried cells or novel juice products was monitored as follows:

- Total mesophilic counts on Plate Count Agar (PCA) (Condalab, Madrid, Spain) after incubation at 30 °C for 72 h.
- Yeasts/molds counts on Malt Agar (Condalab) after incubation 30 °C for 72 h.
- Clostridia on TSC Agar (Condalab) after anaerobic incubation at 37 °C for 24 h.
- *Enterobacteriacae* on Violet Red Bile Glucose Agar (V.R.B.G.A.) (Condalab) after incubation at 37 °C for 24 h.
- Coliforms on Violet Red Bile Agar (V.R.B.A.) (Condalab) after incubation at 30 °C for 24 h.
- Staphylococci on Baird-Parker Agar (BP) (Condalab) after incubation at 37 °C for 24 h.
- *Salmonella* spp. In X.L.D. agar (LabM, UK) at 37 °C.
- *Escherichia coli* on HarlequinTM Chromogenic Media (Condalab) after incubation at 37 °C for 24 h.
- *Pseudomonas aeruginosa* on Pseudomonas agar base—Pseudomonas CN Agar (VWR International GmbH, USA) after incubation at 37 °C for 40–48 h.
- *Listeria monocytogenes* on L-Palcam agar (LabM) fortified with X144 supplement (VWR) after incubation at 37 °C for 48 h.

### 2.6. Physicochemical Analysis

pH was determined on a pH-300i pH meter (WTW GmbH, Weilheim, Germany). 

Water activity (a_w_) was determined using the HygroLab 3 (Rotronic AG, Basserdorf, Switzerland), according to the manufacturer’s guidelines. 

### 2.7. Minor Volatiles 

Samples of novel juice products (20 g) were analyzed for minor volatiles content using the HS-SPME GC/MS technique [6890N GC, 5973NetworkedMS MSD (Agilent Technologies, Santa Clara, CA, USA)], as previously described [42] (Table S1 (Supplementary Materials)).

### 2.8. Preliminary Sensory Evaluation

Novel juice products were assessed for their quality characteristics (aroma, taste, and overall quality) on a 0–5 scale (0: unacceptable, 5: wonderful), as previously reported [43]. All samples were coded, offered in a dark glass under low light and served at 12–15 °C. Between samples, tasters were given water and crackers. 

### 2.9. Statistical Analysis 

All data were analyzed statistically using Analysis of Variance (ANOVA) through Statistica (v.12.0, StatSoft, Tulsa, OK, USA). Significant differences (*p* < 0.05) were determined with the Bonferroni correction.

Component Analysis (PCA) was performed using XLSTAT 2015.1 (Addinsoft, Paris, France).

## 3. Results and Discussions

### 3.1. Storage of Freeze-Dried L. rhamnosus OLXAL-1 Cultures

Initially, free and immobilized *L. rhamnosus* OLXAL-1 cultures (previously evaluated for their antidiabetic properties [8]) were prepared (in wet and freeze-dried form) and their survival rate was monitored. High levels of immobilized cells, ≥ 9 log cfu/g, were recorded in both wet and freeze-dried *L. rhamnosus* OLXAL-1 cultures on apple pieces. In general, during storage for 30 days (Table 1), both free and immobilized *L. rhamnosus* OLXAL-1 cell levels were significantly (*p* < 0.05) affected by the state of the culture (wet or freeze-dried), the storage temperature (4 °C or 20 °C) and the storage duration.

During storage at 20 °C for 30 days, freeze-dried immobilized *L. rhamnosus* OLXAL-1 cells exhibited significantly (*p* < 0.05) higher survival rates (78.8%) than the corresponding freeze-dried free cells (70.5%). In contrast, wet free *L. rhamnosus* OLXAL-1 cells showed 0% survival rate during storage at 20 °C for 30 days, while in the case of the wet immobilized *L. rhamnosus* OLXAL-1 culture, the presence of yeasts/molds was detected (data not shown). During storage at 4 °C for 30 days, the highest (*p* < 0.05) survival rate for immobilized cells on apple pieces was recorded in the case of freeze-dried *L. rhamnosus* OLXAL-1 culture (88.5%), while the survival rate of wet immobilized cultures was diminished down to 0% by day 30 and yeasts/molds were also detected. In the case of freeze-dried free *L. rhamnosus* OLXAL-1 cultures, significantly higher levels were detected (survival rates > 98% recorded) compared to 20 °C, as expected. Similar survival rates of immobilized lactic acid bacteria (LAB) compared to free cultures have also been recently documented during storage at room or refrigeration temperatures [41]. In the same study, a positive effect of immobilization was also observed on the maintenance of cell viability during storage (for 180 days), which resulted in higher survival rates of the freeze-dried immobilized cultures on natural carriers (zea flakes and pistachios) in comparison to the free cultures. Regarding storage, low temperatures are known to prolong cell survival and are thus strongly preferred [44,45], but the nature of the carrier should not be neglected, as it may affect the cell viability throughout the final products’ shelf life and survival [46]. Nevertheless, the possibility of the viability of probiotic cultures (either in free form or immobilized on food ingredients) during long-term storage relying on strain-specific characteristics cannot be excluded [41,47]. 

In addition to the determination of viable *L. rhamnosus* OLXAL-1 cell levels during storage, water activity (a_w_) (Table 1) and moisture levels (Table 1) were also monitored. In general, water activity and moisture levels are key factors that affect both the shelf life of food products and the viability of probiotic cells during storage [48,49]. In particular, it has been reported that for long-term storage of dried probiotic cells, the values of water activity and moisture content are recommended to be < 0.25 and 10%, respectively [48]. For both free and immobilized *L. rhamnosus* OLXAL-1 cells, the lowest levels of moisture and a_w_ were recorded when freeze-drying was applied. This result is in accordance with the higher survival rates recorded in both freeze-dried free and immobilized *L. rhamnosus* OLXAL-1 cells on apple pieces compared to wet cells.

### 3.2. Viability of L. rhamnosus OLXAL-1 Cells in Novel Functional Grape Juice Products

**Figure 1:**
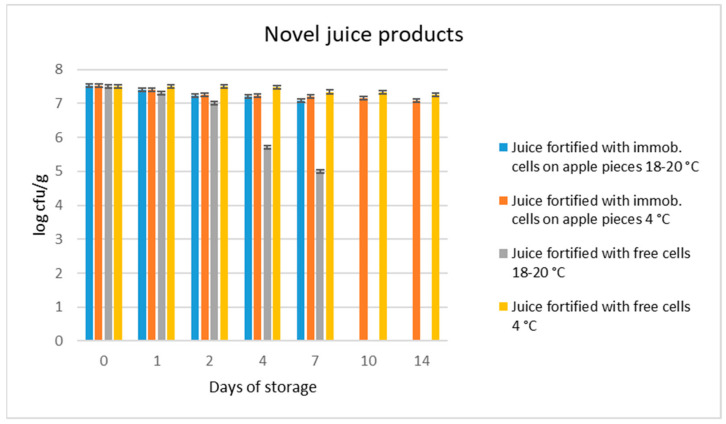
Populations (log cfu/g) of free or immobilized *L. rhamnosus* OLXAL-1 on apple pieces in novel functional juices after 2 weeks storage at 20 °C or 4 °C.

Freeze-dried immobilized cells on apple pieces were directly added in grape juice, reaching a final proportion of 20% *w*/*w* in the reconstituted novel product. Likewise, freeze-dried free cells were directly added in grape juice and served as controls. Samples of both products (containing free or immobilized *L. rhamnosus* OLXAL-1 cells) were then stored at room (20 °C) or refrigeration temperature (4 °C). The microbial stability alongside the effect of storage temperature on any product represents an important aspect of the food industry [16,50], and thus *L. rhamnosus* OLXAL-1 counts were monitored at frequent intervals (Figure 1). 

Initial levels of both free and immobilized *L. rhamnosus* OLXAL-1 cells in both grape juice products were ~7.5 log cfu/g. At ambient temperature, counts of free *L. rhamnosus* cells decreased significantly (*p* < 0.05), while levels of immobilized *L. rhamnosus* cells on apple pieces remained significantly (*p* < 0.05) higher (> 7 log cfu/g) after 4 days of storage. This could be attributed directly to cell immobilization which is well known to protect microbial cells against stresses induced by food production processes [16], resulting in maintenance [51,52,53] or in some cases even enhancement of their counts [32].

In contrast, refrigerated storage resulted in cell loads > 7 log cfu/g for both products (with free and immobilized cells) for up to 14 days, in accordance with previous studies on non-fermented probiotic grape juices [22,24]. In this way, populations > 10^9^ cfu were achieved in a daily product serving (200 mL of juice) [54], thus complying with the minimum recommended concentration needed, in order to confer beneficial health effects on the consumer [9,55]. However, after 14 days of storage, yeasts/molds populations were detected (at concentrations < 3 log cfu/g) and no other data were collected. This is not abnormal, as fruit juices are known to be susceptible to yeasts/molds contamination, despite their acidic environment [56]. Other than that, no changes were recorded on the pH values of our samples (4.3 ± 0.1) throughout storage (in room or refrigeration temperatures), thus indicating a high buffering effect of the novel juice products [57]. 

Notably, at all other time points (up to 10 days), no spoilage or pathogenic microorganisms were detected. In general, the conditions of the raw concentrated grape juice (high osmotic pressure, reduced water activity, etc.) do not favor the survival of pathogens. In some cases, adaptation may occur and for that reason a full screening is officially recommended [56]. However, such a result was not observed in our study, thus implying an extended shelf life for the novel juices (typically 1–5 days) [58], a feature that could surely be exploited by the food industry.

### 3.3. Resistance of Fortified Juices to Microbial Contamination

**Figure 2:**
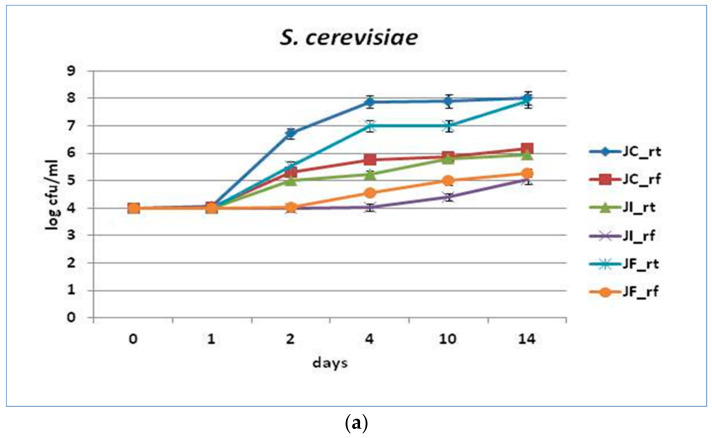

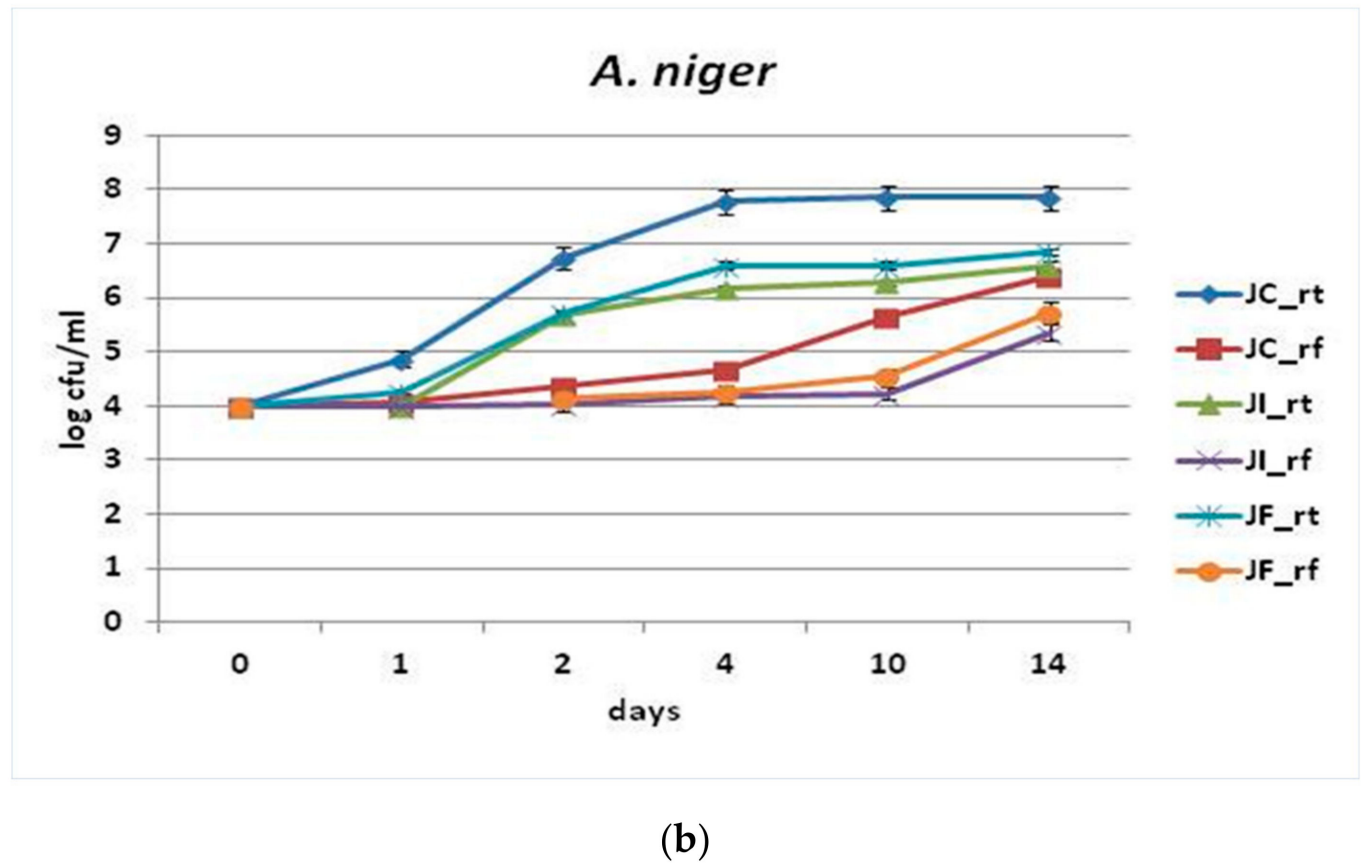
Effect of free or immobilized *L. rhamnosus* OLXAL-1 on apple pieces in novel functional juices deliberately spiked with (**a**) *S. cerevisiae*, or (**b**) *A. niger*, after 2 weeks of storage at 20 °C (rt) or 4 °C (rf). JC: control; JI: Juice with immobilized cells; JF: Juice with free cells.

Possible resistance to microbial spoilage of grape juice containing freeze-dried free or immobilized *Lacticaseibacillus rhamnosus* OLXAL-1 cells on apple pieces after deliberate spiking with *Saccharomyces cerevisiae* or *Aspergillus niger* was investigated (Figure 2). Deliberate contamination of juices fortified with freeze-dried immobilized cells with *S. cerevisiae* or *A. niger* cells resulted in significant growth limitation both at room and refrigeration temperatures compared to the unfortified products, thus exhibiting an antagonistic effect against the spoilage microbes [59,60]. Significant differences on microbial growth, especially in the case of *S. cerevisiae* at 20 °C, were observed between juices with freeze-dried immobilized cells and juices with free cells. In any case, the positive effect of probiotic cultures, as well as the enhanced resistance of immobilized cells against spoilage have previously been reported [61]. These results were also in accordance with a previously published study [8], where *L. rhamnosus* OLXAL-1 cell free supernatant (CFS) exhibited strong inhibitory activity against *S. cerevisiae* and *A. niger*. Notably, no other spoilage or pathogenic microorganisms were detected and no pH changes were recorded, as mentioned above. Despite previous efforts investigating the use of probiotics against food-spoilage microorganisms [59,60], to the best of our knowledge, none have implicated the use of immobilized cultures against deliberate spiking of juice products.

### 3.4. Minor Volatiles Determination and Chemometrics

Novel juice products were subjected to HS-SPME GC/MS analysis, in order to determine minor volatiles responsible for aroma (Table S1). Keynote compounds, normally present in grape juice, like ethyl acetate (known for contributing to aromatic complexity), 2-phenylethyl acetate (known for its rose aroma), furfural (known for adding notes of freshly baked bread), 2-phenylethanol (known for rose scents), as well as 2- and 3-methyl-1-butanol (known for adding whiskey malt notes and a burnt aroma) were detected in all juice products [62,63,64,65,66,67,68]. Other compounds like hexanal (grassy, green) and (E)-2-hexenal (green) are usually linked to the apples (immobilization carrier) and were identified only in the products fortified with immobilized cells [69]. However, their presence could also be a result of microbial metabolism and their inhibitory effect against *Saccharomyces* and *Aspergillus* species has been previously well documented [70,71,72,73,74].

Linalool and α-terpineol (known to add lime tree notes and lilac aroma, respectively), usually found in grape berries [68], were also identified in all juice products, without significant differences in their concentration, in most cases. In general, the existence of terpenes in the juice may be associated with an enhancement of the product’s shelf life, as their antimicrobial [75,76] and/or antioxidant role [77] has been well documented, or even be associated with significant health benefits for the consumer [78].

**Figure 3:**
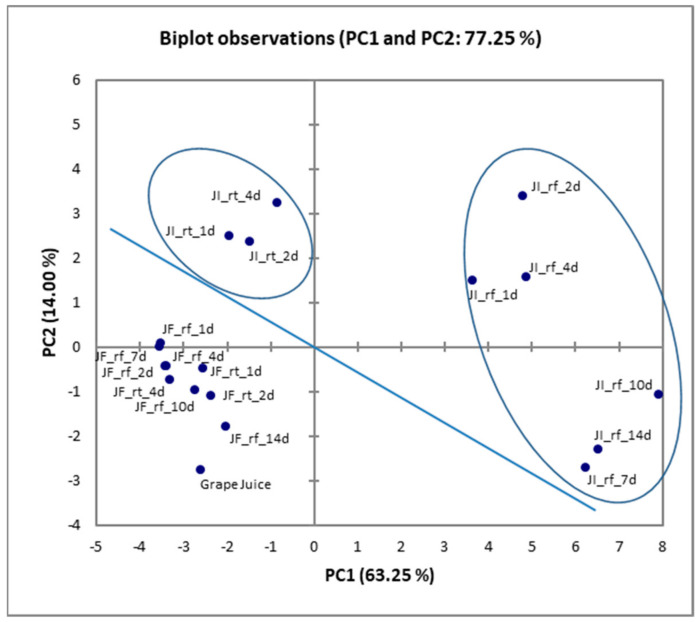
Principal component analysis (PCA) plot of minor volatiles isolated by novel juice products fortified with freeze-dried *L. rhamnosus* OLXAL-1 cells. JF: juice fortified with free cells, JI: juice fortified with immobilized cells on apple pieces. The storage temperature is indicated as rt (room temperature) or rf (refrigeration), while the storage days are shown at the end of the sample code.

Principal Component Analysis (PCA) applied to HS-SPME results revealed that the nature of the freeze-dried cells (free or immobilized) used, as well as the storage conditions (room or refrigeration temperature) significantly affected (*p* < 0.05) the aromatic characteristics of the novel juice products (Figure 3). Specifically, juice products fortified with freeze-dried free *L. rhamnosus* OLXAL-1 cells were gathered in the lower left part of the diagram, while juice products fortified with freeze-dried immobilized cells were concentrated in the upper and right regions, respectively. In addition, the juice products fortified with freeze-dried immobilized *L. rhamnosus* OLXAL-1 cells formed two distinct subgroups in the diagram, depending on the storage temperature applied (room temperature or refrigeration temperature). In particular, storage of samples fortified with freeze-dried immobilized cells at 4 °C caused a concentration increase in most volatiles [79] and resulted in the highest (*p* < 0.05) total volatiles content (Table S1) for each timepoint.

### 3.5. Preliminary Sensory Evaluation

Despite the importance of factors like the physicochemical/microbial stability and the product’s nutritional value, the sensory characteristics play an important role in the consumers’ acceptability [80]. Thus, all new juice products were evaluated regarding their aroma (fruity, floral, wine-like, caramel, other) and taste (sweet, sour, bitter, salty) by a mixed panel of 20 untrained tasters. According to the results (Table 2), all products were characterized by a predominant wine-like/fruity (grape) aroma, as a result of the esters, alcohols and terpenes found in the juice (Table S1). Distinct apple notes were distinct in the case of products containing immobilized cells, deriving from characteristic compounds found in the immobilization carrier (apple pieces). The taste was strongly sweet in all cases, as no juice fermentation occurred, with a pleasant aftertaste and a refreshing feeling. Both aroma and taste were described as “fully natural” by the tasters in all samples and no off notes were detected.

At the same time, feedback on the products’ novelty was gathered. Notably, products with freeze-dried immobilized *L. rhamnosus* OLXAL-1 cells on apple pieces emerged in the testers’ preference, most likely due to the originality of the product. After all, the appearance of a product (color, shape, size, etc.) is known to constitute the basic characteristic responsible for the product’s identification and selection, and strongly affects concepts like craveability and appetite [81]. In our case, juice products containing immobilized cells were significantly preferred (*p* < 0.05) against the juice products with free cells and gathered significantly higher scores regarding the overall evaluation. Notably, the serving of freeze-dried apple pieces (containing immobilized cells) and grape juice in different containers, with the testers’ direct involvement in the process of reconstituting the final juice product, was characterized as highly interesting compared to the directly served juice product with freeze-dried free cells. As a matter of fact, attributes like the style of presentation are sought to be exploited, in terms of sensory marketing, as they can influence the consumers’ perception, judgment and behavior, affect their satisfaction and result in indirect product promotion [1,82].

## 4. Conclusions

A novel grape juice product fortified with freeze-dried free or immobilized *L. rhamnosus* OLXAL-1 cells (previously evaluated for their antidiabetic properties) was developed. The use of immobilized cells on apple pieces resulted in significantly higher counts compared to the free cells at 20 °C, while refrigerated storage resulted in cell loads > 7 log cfu/g for both products for up to 10 days, thus achieving populations > 10^9^ cfu per share. *L. rhamnosus* OLXAL-1 cells resulted in significant growth limitation of *S. cerevisiae* and *A. niger* in deliberately spiked products, exhibiting an antagonistic effect. Minor volatiles detected by HS-SPME GC/MS were mostly linked to either the grape juice or the immobilization carrier (apple pieces), while the nature of the freeze-dried cells (free or immobilized) and the storage conditions (room or refrigeration temperature) significantly affected the aromatic characteristics of the novel juice products. Notably, all novel juice products werecharacterized as highly original and were accepted during the preliminary sensory evaluation. 

In conclusion, data supporting the development of a novel functional juice containing freeze-dried immobilized *L. rhamnosus* OLXAL-1 cells on apple pieces with great market potential are presented. The wild-type culture used may serve as a biopreservative agent to prolong the shelf life of juices and prevent spoilage. However, more research is still required to verify their health-promoting effects in humans and their effectiveness in industrial production. 

## Figures and Tables

**Table 1:** Survival rate (%), water activity (a_w_) and moisture content (%) of freeze-dried free and immobilized *L. rhamnosus* OLXAL-1 cells after short-term storage.

Storage Time (Days)	Wet Immobilized Cells on Apple pieces		Wet Free Cells		Freeze-Dried Immobilized Cells on Apple Pieces		Freeze-Dried Free Cells	
	20 °C	4 °C	20 °C	4 °C	20 °C	4 °C	20 °C	4 °C
	Survival rate (%)							
15	88.73 ± 0.40	96.65 ± 0.34	55.33 ± 1.11	85.66 ± 0.91	79.84 ± 0.02	95.13 ± 0.90	90.46 ± 0.29	99.03 ± 0.45
30	0 *	0 *	0	27.54 ± 0.67	78.84 ± 0.89	88.45 ± 0.75	70.50 ± 1.41	98.07 ± 0.15
	Water activity (a_w_)							
0	0.911 ± 0.011		0.919 ± 0.004		0.202 ± 0.005		0.076 ± 0.006	
15	0.900 ± 0.001	0.905 ± 0.03	0.900 ± 0.002	0.901 ± 0.001	0.299 ± 0.003	0.277 ± 0.001	0.140 ± 0.001	0.087 ± 0.002
30	- *	- *	0.898 ± 0.003	0.886 ± 0.001	0.338 ± 0.001	0.313 ± 0.001	0.147 ± 0.002	0.090 ± 0.001
	Moisture content (%)							
0	85.75 ± 0.75		53.09 ± 0.11		10.99 ± 1.27		2.90 ± 0.02	
15	86.62 ± 0.25	87.07 ± 0.91	40.94 ± 0.12	53.71 ± 1.26	16.76 ± 1.80	14.41 ± 0.18	4.09 ± 0.02	5.32 ± 0.28
30	- *	- *	30.13 ± 0.66	54.55 ± 0.23	19.42 ± 0.96	17.72 ± 0.35	6.47 ± 0.21	5.64 ± 0.33

* Presence of molds.

**Table 2:** Sensory evaluation of novel juice products fortified with free or immobilized freeze-dried *L. rhamnosus* OLXAL-1 cells.

Sensory Evaluation Attribute	JI	JF
Aroma	3.4 ± 0.5	3.2 ± 0.4
Taste	3.8 ± 0.4	3.8 ± 0.8
Product novelty (appearance, juice color, serving, etc.)	4.8 ± 0.3	2.9 ± 0.4
Overall evaluation	3.8 ± 0.7	3.1 ± 0.4

## Data Availability

The data presented in this study are available on request from the corresponding author. The data are not publicly available due to restrictions of the funding authorities.

## Supplementary Materials

The following supporting information can be downloaded at: https://www.mdpi.com/article/10.3390/microorganisms11030646/s1, Table S1: Minor volatiles (mg/L) detected by HS-SPME GC/MS analysis in grape juice products fortified with freeze-dried free or immobilized *L. rhamnosus* OLXAL-1 cells, after storage at room (20 °C) or refrigeration (4 °C) temperature.
