# Supplementary material for: Novel Functional Grape Juices Fortified with Free or Immobilized Lacticaseibacillus rhamnosus OLXAL-1
Source: Microorganisms. 2023 Mar 2;11(3):646. doi: 10.3390/microorganisms11030646 (PMC10051719; doi:10.3390/microorganisms11030646)
Supplement: Supplementary file 1 [file microorganisms-11-00646-s001.zip › microorganisms-2216918-supplementary/Nikolaou et al_Supplementary_R.pdf]

Table S1. Minor volatiles (mg/L) detected by HS-SPME GC/MS analysis in grape juice products fortified with freeze-dried free or immobilized *L. rhamnosus* OLXAL-1 cells, after storage at room (20 ˚C) or refrigeration (4 ˚C) temperature.

**Compounds detected**			**Storage at room temperature (20 ˚C)**						**Storage at refrigeration temperature (4 ˚C)**									
	KI	**Grape Juice**	**JF_1d**	**JF_2d**	**JF_4d**	**JI_1d**	**JI_2d**	**JI_4d**	**JF_1d**	**JF_2d**	**JF_4d**	**JF_7d**	**JF_10d**	**JI_1d**	**JI_2d**	**JI_4d**	**JI_7d**	**JI_10d**
Ethyl acetate	<700	<0.1	<0.1	<0.1	<0.1	0.1±0.1	0.1±0.1	0.1±0.1	<0.1	<0.1	<0.1	<0.1	<0.1	0.1±0.1	0.1±0.1	0.1±0.1	0.1±0.1	0.1±0.1
2-methyl-1-propanol	<700	<0.1	<0.1	<0.1	<0.1	<0.1	<0.1	0.1±0.1	<0.1	<0.1	<0.1	<0.1	<0.1	0.1±0.1	0.1±0.1	0.1±0.1	0.1±0.1	0.1±0.1
3-methyl-butanal	<700	n.d.	0.1±0.1	0.1±0.1	<0.1	0.1±0.1	0.1±0.1	0.1±0.1	0.1±0.1	0.1±0.1	0.1±0.1	0.1±0.1	<0.1	0.1±0.1	0.2±0.1	0.2±0.1	0.1±0.1	0.1±0.1
2-methyl-butanal	<700	n.d.	<0.1	<0.1	<0.1	0.1±0.1	0.1±0.1	0.1±0.1	0.1±0.1	0.1±0.1	0.1±0.1	<0.1	<0.1	0.1±0.1	0.1±0.1	0.1±0.1	<0.1	<0.1
3-methyl-1-butanol	721	0.3±0.1	0.2±0.1	0.2±0.1	0.2±0.1	0.3±0.1	0.4±0.1	0.4±0.2	0.2±0.1	0.2±0.1	0.2±0.1	0.3±0.1	0.3±0.1	0.4±0.1	0.5±0.2	0.5±0.1	0.5±0.2	0.5±0.1
2-methyl-1-butanol	722	0.2±0.1	0.2±0.1	0.2±0.1	0.1±0.1	0.3±0.1	0.3±0.1	0.3±0.2	0.2±0.1	0.2±0.1	0.2±0.1	0.2±0.1	0.2±0.1	0.2±0.1	0.4±0.1	0.3±0.1	0.2±0.1	0.5±0.2
Hexanal	800	n.d.	n.d.	n.d.	n.d.	0.1±0.1	0.2±0.1	0.2±0.1	n.d.	n.d.	n.d.	n.d.	n.d.	0.7±0.1	0.7±0.1	0.8±0.1	0.7±0.2	1.0±0.2
Furfural	835	0.9±0.4	3.0±0.1	3.2±0.3	3.1±0.2	2.0±0.2	2.3±0.1	2.4±0.2	1.0±0.1	1.2±0.1	1.1±0.1	0.9±0.1	1.2±0.1	6.0±0.7	7.8±0.5	7.0±0.4	7.3±0.8	8.1±0.4
(E)-2-hexenal	854	n.d.	n.d.	n.d.	n.d.	0.3±0.1	0.3±0.1	0.3±0.1	n.d.	n.d.	n.d.	n.d.	n.d.	0.5±0.1	0.6±0.1	0.6±0.1	0.7±0.1	1.0±0.1
(E)-2-hexen-1-ol	857	n.d.	n.d.	n.d.	n.d.	n.d.	n.d.	n.d.	n.d.	n.d.	n.d.	n.d.	n.d.	0.1±0.1	0.1±0.1	0.1±0.1	0.2±0.1	0.2±0.1
1-hexanol	861	n.d.	n.d.	n.d.	n.d.	0.1±0.1	0.2±0.1	0.3±0.1	n.d.	n.d.	n.d.	n.d.	n.d.	0.3±0.1	0.2±0.1	0.4±0.1	0.4±0.1	0.5±0.1
1-(2-furanyl)-ethanone	902	n.d.	<0.1	0.1±0.1	0.1±0.1	0.1±0.1	0.1±0.1	0.2±0.1	0.1±0.1	0.1±0.1	0.1±0.1	0.1±0.1	0.1±0.1	0.4±0.1	0.6±0.3	0.3±0.1	0.4±0.1	0.4±0.1
5-methyl-2-furancarboxaldehyde	960	0.2±0.1	0.3±0.1	0.3±0.1	0.4±0.2	0.2±0.1	0.3±0.1	0.3±0.1	0.2±0.1	0.3±0.1	0.4±0.2	0.2±0.1	0.6±0.1	1.0±0.2	0.9±0.1	1.0±0.2	1.2±0.1	2.0±0.1
6-methyl-5-hepten-2-one	988	n.d.	n.d.	n.d.	n.d.	0.3±0.1	0.6±0.1	0.4±0.1	n.d.	n.d.	n.d.	n.d.	n.d.	0.8±0.2	0.6±0.1	1.2±0.1	2.0±0.1	3.1±0.5
Benzeneactealdehyde	1047	n.d.	0.2±0.1	0.2±0.1	0.2±0.1	0.1±0.1	0.1±0.1	0.1±0.1	0.1±0.1	0.1±0.1	0.1±0.1	0.1±0.1	0.2±0.1	0.4±0.1	0.3±0.1	0.4±0.1	0.6±0.2	0.5±0.2
3-methyl-benzaldehyde	1059	n.d.	<0.1	<0.1	0.1±0.1	<0.1	<0.1	<0.1	n.d.	n.d.	n.d.	n.d.	n.d.	n.d.	n.d.	n.d.	n.d.	n.d.
Linalool oxide	1073	n.d.	n.d.	n.d.	n.d.	n.d.	n.d.	n.d.	0.1±0.1	0.1±0.1	0.1±0.1	0.1±0.1	0.2±0.1	0.3±0.1	0.2±0.1	0.3±0.2	0.4±0.1	0.5±0.2
3,7-dimethyl-1,6-octadien-3-ol (linalool)	1123	0.5±0.1	0.4±0.2	0.5±0.1	0.4±0.1	0.1±0.1	0.2±0.1	0.1±0.1	0.2±0.1	0.3±0.1	0.3±0.1	0.2±0.1	0.3±0.1	0.5±0.2	0.3±0.1	0.6±0.2	1.3±0.4	0.6±0.1
2-phenylethanol	1133	0.5±0.1	0.2±0.1	0.4±0.1	0.1±0.1	0.1±0.1	0.2±0.1	0.2±0.1	0.1±0.1	0.1±0.1	0.1±0.1	0.1±0.1	0.3±0.1	0.4±0.1	0.3±0.1	0.7±0.1	1.0±0.3	0.9±0.2
α-Terpineol	1190	0.9±0.1	0.9±0.2	0.9±0.1	0.8±0.1	0.5±0.1	0.4±0.1	0.5±0.1	0.2±0.1	0.4±0.1	0.3±0.1	0.2±0.1	0.3±0.1	1.2±0.1	1.0±0.1	1.5±0.2	1.9±0.2	2.0±0.4
Diethyl butanedioate	1191	0.1±0.1	n.d.	n.d.	n.d.	n.d.	n.d.	n.d.	n.d.	n.d.	n.d.	n.d.	n.d.	<0.1	<0.1	<0.1	0.1±0.1	0.1±0.1
1,3-bis(1,1-dimethylethyl)-benzene	1258	0.1±0.1	0.1±0.1	0.1±0.1	0.1±0.1	0.1±0.1	0.1±0.1	0.1±0.1	<0.1	<0.1	<0.1	<0.1	0.1±0.1	0.3±0.1	0.4±0.1	0.4±0.2	0.4±0.2	0.5±0.1
2-phenylethyl acetate	1263	1.0±0.2	0.6±0.1	0.7±0.2	0.6±0.2	0.2±0.1	0.3±0.1	0.3±0.1	0.2±0.1	0.3±0.1	0.2±0.1	0.2±0.1	0.2±0.1	1.5±0.2	2.0±0.3	1.2±0.2	1.3±0.1	1.4±0.1

Hexyl hexanoate	1380	n.d.	n.d.	n.d.	n.d.	0.3±0.1	0.3±0.1	0.3±0.1	n.d.	n.d.	n.d.	n.d.	n.d.	0.2±0.1	0.5±0.2	0.3±0.1	0.1±0.1	0.4±0.2
2,4-bis(1,1-dimethylethyl)-phenol	1516	n.d.	0.3±0.1	0.3±0.1	0.3±0.1	0.1±0.1	0.1±0.1	0.1±0.1	<0.1	<0.1	<0.1	<0.1	0.1±0.1	0.2±0.1	0.4±0.1	0.2±0.1	0.2±0.1	0.2±0.1
Total		4.7	6.6	7.2	6.5	5.5	6.7	6.9	2.8	3.5	3.3	2.8	4.1	15.8	18.3	18.3	20.8	24.7

KI: Kovats retention index; n.d.: not detected; JF: juice fortified with free cells, JI: juice fortified with immobilized cells on apple pieces. The storage days are shown at the end of the sample code.
